# Synthesis of Antibacterial Nisin–Peptoid Hybrids Using Click Methodology

**DOI:** 10.3390/molecules23071566

**Published:** 2018-06-28

**Authors:** Hannah L. Bolt, Laurens H. J. Kleijn, Nathaniel I. Martin, Steven L. Cobb

**Affiliations:** 1 Center for Global Infectious Diseases, Department of Chemistry, Durham University, South Road, Durham DH1 3LE, UK; bolth@medimmune.com; 2 Department of Medicinal Chemistry and Chemical Biology, Utrecht Institute for Pharmaceutical Sciences, Utrecht University, Universiteitsweg 99, 3584 CG Utrecht, The Netherlands; l.h.j.kleijn@uu.nl

**Keywords:** peptoids, peptoid–peptide hybrid, nisin, antibacterial, alkyne-azide click reactions

## Abstract

Antimicrobial peptides and structurally related peptoids offer potential for the development of new antibiotics. However, progress has been hindered by challenges presented by poor in vivo stability (peptides) or lack of selectivity (peptoids). Herein, we have developed a process to prepare novel hybrid antibacterial agents that combine both linear peptoids (increased in vivo stability compared to peptides) and a nisin fragment (lipid II targeting domain). The hybrid nisin–peptoids prepared were shown to have low micromolar activity (comparable to natural nisin) against methicillin-resistant *Staphylococcus aureus.*

## 1. Introduction

The rapid emergence of antibiotic resistance against commonly used frontline treatments poses a serious threat to global health and has emphasized the need for novel antimicrobials to be developed. This urgency is highlighted by increasing academic and industrial interest in the field and the establishment of enterprises such as the 2015 Ross Fund and the 10 × ‘20 Initiative both of which encourage the delivery of new antimicrobial drug classes [1,2].

**Figure 1:**
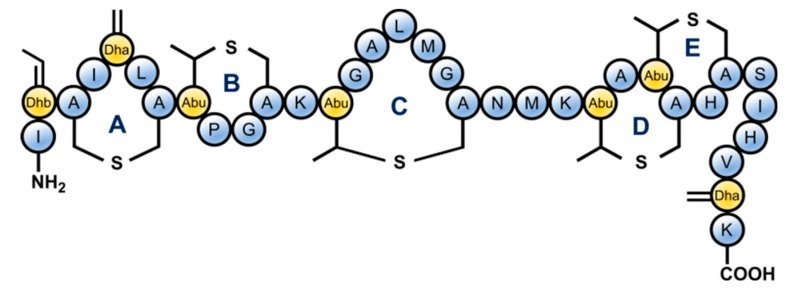
The structure of nisin. Classified as a lantibiotic due to the presence of unusual lanthionine rings in its structure, nisin also contains unsaturated amino acids introduced by posttranslational modifications: Dhb = dehydrobutyrine, Dha = dehydroalanine and Abu = aminobutyric acid.

Nisin is a polycyclic peptide produced by several strains of the Gram-positive bacterium *Lactococcus lactis* and is an antimicrobial with nanomolar activity against other Gram-positive bacteria [3,4]. Due to its potent antibacterial activity and negligible toxicity, nisin (Figure 1) is frequently used as a food preservative and it provides a potential scaffold for the development of novel antibacterial agents [3,4]. Nisin contains five lanthione bridges which help impart a conformation that is key to its antibacterial properties [5,6]. In particular the A/B ring system at nisin’s N-terminus has been shown to bind to lipid II, which is a crucial membrane component used in bacterial cell wall synthesis. Following lipid II binding, nisin can insert into the bacterial plasma membrane and create pores that cause cell leakage ultimately leading to cell death [5,7,8,9,10,11,12].

Given its selective mode of action, nisin provides an excellent starting point for the design of new antibiotics. However, due to nisin’s peptide-based structure, the clinical development of the full-length peptide sequence remains challenging. Specifically, the high susceptibility of nisin towards proteolytic degradation in vivo has presented a major hurdle in its use as a general antibiotic [10,13]. Efforts to overcome stability issues via the total chemical synthesis or semi-synthesis of nisin analogues with improved properties have only had limited success [14,15,16].

Early studies in the area demonstrated that C-terminally truncated nisin peptides that still contain an intact A/B ring system (nisin^A/B^) can still display low antibacterial activity [17]. The aforementioned results, led to numerous truncated nisin peptides being biologically evaluated, including recently promising semi-synthetic nisin-lipopeptides [9]. 

**Figure 2:**
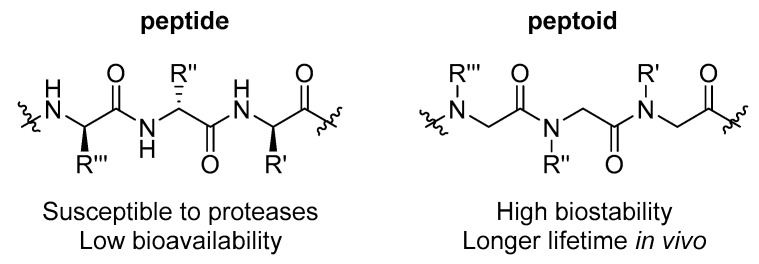
Representative structure of the backbone of a peptide compared to a peptoid. In peptoids, the side chain ‘R’ is moved to the nitrogen of the amide backbone, causing a substantial increase in chemical and biological stability.

A strategy that is more commonly being utilized to stabilize peptide-based therapeutics is the synthesis of stable peptidomimetics such as peptoids (*N*-substituted glycines) (Figure 2) [18,19,20]. Peptoids have shown activity as antibacterial [21,22,23,24,25,26,27,28], antifungal [29,30,31] and antiparasitic [32,33,34,35] compounds and most importantly they display increased resistance to protease action compared to α-peptides [31,36]. In particular, peptoids have demonstrated antibacterial properties against a range of clinically relevant Gram negative and Gram positive bacteria, with activities that equal or surpass those achieved by many antimicrobial peptides (AMPs) [21,22,23,24,25,26,27,28]. Peptoids are also suspected to disrupt cell membranes non-specifically to achieve their antimicrobial effect, with similar modes of actions to simple AMPs [21,37]. Given this, peptoids display activity against many drug-resistant bacteria.

Building on from this, we wished to investigate whether we could prepare semi-synthetic nisin analogues (e.g., peptide–peptoid hybrids) with comparable antibacterial activity to full-length nisin. To this end, we report a new class of peptide–peptoid hybrids that could be used in the development of novel antimicrobials. By linking short linear peptoids with the truncated nisin^A/B^ ring fragment, we were able to access molecules that have similar antibacterial activity against methicillin-resistant *S. aureus* compared to full-length nisin. 

## 2. Results and Discussion

Nisin^A/B^ is obtained from the chemo-enzymatic degradation of full-length nisin [38] and this fragment can then be modified at the C terminus by addition of an azide functionality [9]. The azide provides a convenient handle for ligation to alkynes via a copper-catalyzed click cycloaddition [39]. The click reaction between an azide and a terminal alkyne is compatible with a range of conditions, tolerates a broad range of chemical functionalities and provides a robust strategy for chemical ligations. Furthermore, as an isostere of the amide bond, the triazole formed by click reactions provides a biocompatible linker that readily associates with biological targets through hydrogen bonding and dipole interactions [39]. Additionally, the copper (I)-catalyzed click reaction can be performed under aqueous conditions at room temperature, so is well suited for use with peptide-based reagents [40]. For these reasons, the copper-catalyzed click reaction is ideal for the ligation of azide-functionalzsed nisin with terminal alkynyl peptoid sequences [41].

**Scheme 1:**
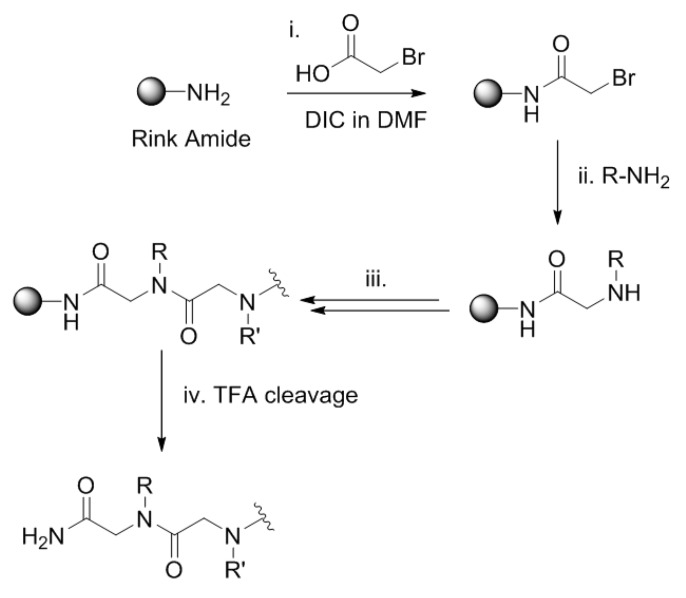
Scheme to show the solid-phase, submonomer synthesis of peptoids: (i) acylation using bromoacetic acid and DIC in DMF, 20 min, RT; (ii) displacement with primary amine, 1.5 M, RT, 60 min; (iii) repeated acylation and displacement to complete sequence; (iv) acidic TFA cleavage and deprotection of peptoid.

Two linear peptoid sequences (peptoid **1a** (*N*ae*N*spe*N*spe)_4_ and **1b** [(*N*Lys*N*pfb*N*pfb)(*N*Lys*N*spe*N*spe)]_2_) were chosen as they had shown promising antibacterial activities with MICs below 2 µM against *S. aureus*. Peptoids **1a** and **1b** are both 15 residues in length; i.e., the 12 residue parent sequence followed by two additional *N*spe monomers to lengthen the chain between the last cationic residue and the N terminal *N*prp monomer required for the click reaction. Peptoids **2a** *N*prp*N*spe*N*spe(*N*ae*N*spe*N*spe)_4_ and **2b** *N*prp*N*spe*N*spe[(*N*Lys*N*pfb*N*pfb)(*N*Lys*N*spe*N*spe)]_2_ were prepared via the submonomer procedure for the solid phase synthesis of peptoids, which utilizes successive acylation and displacement cycles (see Scheme 1). An alkyne tail was added to the sequence using propargylamine (*N*prp) under standard coupling conditions [34,42,43]. Peptoids **2a** and **2b** were then cleaved from the resin and purified by RP-HPLC prior to the click reactions being performed.

**Scheme 2:**
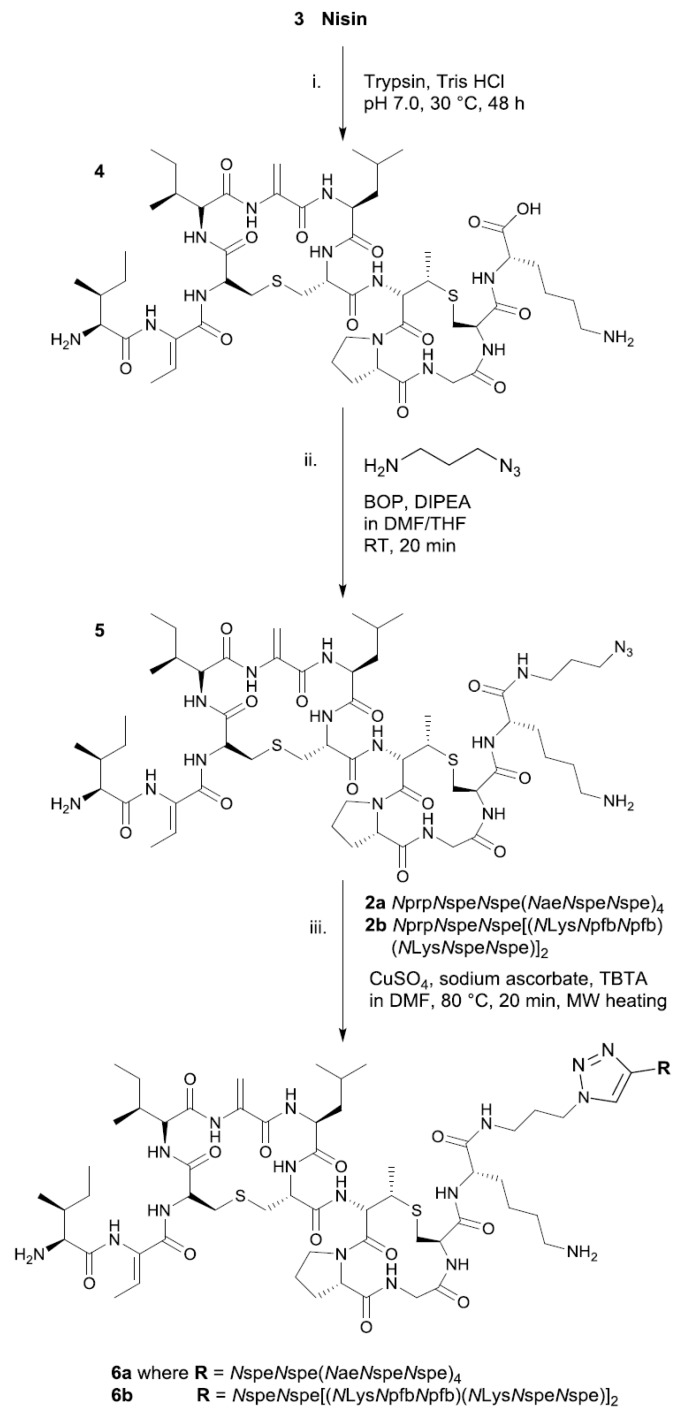
Semi-synthesis of nisin^A/B^–peptoid hybrids using (i) digestion of nisin to the nisin^A/B^ ring; (ii) addition of azide functionality to nisin^A/B^; (iii) Cu(I) catalyzed click reaction with alkyne functionalized peptoid sequences.

Nisin (**3**) was digested using trypsin and the fragment containing the A/B ring system (**4**) isolated by preparative HPLC. The nisin^A/B^ was then coupled to azidopropylamine and a second RP-HPLC purification undertaken to yield the truncated nisin with an azide linker (compound **5** in Scheme 2) [9].

Ligation of nisin^A/B^-azide and the alkyne-tagged peptoids was carried out under microwave irradiation using a copper(I)-catalyzed ‘click’ cycloaddition. The active Cu(I) catalyst is generated from the Cu(II) salt using sodium ascorbate as a reducing agent and with Tris[(1-benzyl-1*H*-1,2,3-triazol-4-yl)methyl]amine (TBTA) as a Cu(I) stabilizing ligand to cleanly afford the expected triazole product [39,40,44]. Completion of the click reaction was observed via LC-MS after 20 min microwave heating at 80 °C. Coupling times were limited to 20 min to avoid unwanted side reactions (i.e., Dha cyclization or lactamization) as previously described [9]. Following a final RP-HPLC purification, the expected conjugates (**6a** and **6b**) were obtained in suitable purity for biological testing. Full methods and the characterization of **6a** and **6b** can be found in the Supplementary Materials.

The antibacterial activity of the nisin–peptoid conjugates **6a** and **6b** were determined against methicillin-resistant *Staphylococcus aureus* USA 300; the predominant strain associated with community-acquired MRSA infection in the United States [45] (see Table 1). The minimum inhibitory concentrations (MICs) were calculated and compared to the peptide antibiotic vancomycin **7** and full-length nisin (**3**).

The parent peptoids without an alkyne linker demonstrated excellent activity against the methicillin-resistant *S. aureus*, with **1a** and **1b** displaying MICs of 2 µM and 2–4 µM respectively. Interestingly, simply functionalizing these peptoid sequences with an N-terminal alkyne reduces their activity by approximately a factor of 2 (**2a**, 4 µM and **2b**, 4–7 µM). The nisin–peptoid hybrids **6a** and **6b** also show diminished antibacterial activity compared to the unconjugated peptoids (**1a** and **1b**). When comparing **6a** to **1a**, the nisin–peptoid conjugate is approximately five-fold less active than the parent peptoid (i.e., MIC 5–10 µM compared to 2 µM) and this reduction in activity is similarly seen when comparing peptoid **6b** and **1b** (MIC 9–18 µM vs. 2–4 µM). However, despite the reduction in activity compared to the peptoids alone, the MIC values for **6a** and **6b** are still in the low micromolar range against a drug resistant bacterium. In addition, **6a** has a MIC similar to that of full-length nisin (**3**), and one that is considerably better than truncated nisin[1,2,3,4,5,6,7,8,9,10,11,12] (**4**).

In order to ascertain whether the decreased activity displayed by the nisin–peptoid conjugates (when compared to their parent peptoid sequences) was accompanied by a beneficial reduction in toxicity, cytotoxicity assays were performed against HaCaT keratinocytes and epithelial HepG2 cells. Results for this testing can be found in the ESI and the data obtained shows that the peptoid sequences with the N terminal alkyne (**2a** and **2b**) were the most cytotoxic to both mammalian cell lines tested (ED_50_ values of <20 µM). The nisin-conjugated peptoid sequences **6a** and **6b** were found to be moderately cytotoxic to both mammalian cell lines tested (e.g., ED_50_ values of 22 µM and 24 µM against HaCaT for **6a** and **6b** respectively). The ED_50_ values obtained also showed that the nisin–peptoid hybrids (**6a** and **6b**) have either a similar level or increased toxicity to the HaCaT cells compared to the parent peptoids **1a** and **1b** (e.g., ED_50_ values of 26 µM and 53 µM against HaCaT for **1a** and **1b** respectively).

## 3. Materials and Methods

### 3.1. Linear Peptoid Synthesis (***1a***, ***1b***, ***2a*** and ***2b***)

Fmoc-protected Rink Amide resin (0.1 mmol, loading 0.82 mmol g^−1^) was swollen in DMF (at least 1 h, overnight preferred, at room temperature) in a 20 mL polypropylene syringe fitted with two polyethylene frits. The resin was deprotected with piperidine (20% in DMF *v/v*, 2 × 20 min) and washed with DMF (3 × 2 mL). The resin was treated with bromoacetic acid (1 mL, 0.6 M in DMF) and DIC (0.20 mL, 50% *v/v* in DMF) for 20 min at room temperature at 400 rpm. The resin was washed with DMF (3 × 2 mL), before the desired amine sub-monomer was added (1 mL, 0.8–2.0 M in DMF) and allowed to react for 60 min at room temperature on the shaker. The resin was again washed with DMF (3 × 2 mL) and the bromoacetylation and amine displacement steps were repeated until the final submonomer had been added and the desired peptoid sequence had been obtained. The resin was shrunk in diethyl ether to remove DMF in preparation for cleavage. Final cleavage from resin was achieved using TFA (95%), H_2_O (2.5%) and TIPS (2.5%). For test cleaves approximately 1 mL of the cleavage cocktail was used and for cleavage from 100 mg resin, approximately 4 mL of the cleavage cocktail was added. The resin was then placed on the shaker at 400 rpm for 45 min and the resin removed by filtration. The cleavage cocktail was removed in vacuo, the crude product precipitated in diethyl ether (45 mL) and the precipitate retrieved by centrifuge for 15 min at 5000 rpm. The ether phase was decanted, the crude product dissolved in a mixture of acidified H_2_O and MeCN and lyophilized. Crude peptoid sequences were purified using RP-HPLC prior to ligation with nisin^A/B^. For further details see the Supplementary Materials.

### 3.2. Amide-Coupled Azide-Nisin^A/B^ (***5***)

Nisin (600 mg, 0.18 mmol) was dissolved in 250 mL Tris buffer (25 mmol, NaOAc, 5 mmol Tris acetate, 5 mmol CaCl_2_, pH 7.0) and the solution cooled on ice for 15 min. Trypsin (50 mg) was added and stirred at room temperature for 15 min. The mixture was then heated to 30 °C for 16 h, then another 50 mg of trypsin was added and after an additional 24 h the reaction was complete by HPLC. The reaction was acidified with HCl (1 M) to pH 4.0 and solvents removed in vacuo. The nisin fragment was isolated by preparative HPLC and product fractions lyophilized to obtain a white powder (80 mg, 39%). Nisin^A/B^ was dissolved in DMF (240 µL). BOP (2 eq.), DIPEA (4 eq.) and azidopropylamine (50 eq.) were added. The reaction was stirred for 20 min then quenched in the appropriate buffer (95% H_2_O, 5% MeCN, 0.1% TFA, 4 mL). The solution was centrifuged for 5 min at 5000 rpm to remove any insoluble material and the supernatant was purified by RP-HPLC. Relevant fractions were collected and analyzed to yield the pure Nisin^A/B^-azide. For further details see the Supplementary Materials.

### 3.3. Click Protocol of Alkyne-Peptoids with Nisin^A/B^-Azide (***6a***, ***6b***)

10× stock solutions of CuSO_4_ (16.2 µmol, 2.59 mg in 1 mL H_2_O), 10× sodium ascorbate (32.4 µmol, 6.42 mg in 1 mL H_2_O) and 10× TBTA (4.1 µmol, 2.18 mg in 1 mL DMF) were freshly prepared. Nisin^A/B^-azide (1 eq., 8.1 µmol, 10 mg) was dissolved in 200 µL DMF and added to the peptoid in the microwave reaction vessel (1 eq., 8.1 µmol). 100 µL of the CuSO_4_ solution (0.2 eq., 1.62 µmol), 100 µL of sodium ascorbate stock (0.4 eq., 3.24 µmol) and 100 µL of the TBTA solution (0.05 eq., 0.41 µmol) were added. The vessel was sealed and heated under microwave power for 20 min at 80 °C. The reaction mixture was diluted in the appropriate buffer (95% H_2_O, 5% MeCN, 0.1% TFA, 4 mL) and purified by RP-HPLC on a Reprospher 100 C8- or C18- Aqua column (10 µm × 250 mm× 20 mm) at a flow rate of 6 mL min^−1^; λ = 214 nm; linear gradient elution 20–80% solvent B over 120 min (where A = 95% H_2_O, 5% MeCN, 0.1% TFA; B = 95% MeCN, 5% H_2_O, 0.1% TFA). Relevant fractions were combined and lyophilized from 1:1 H_2_O:*^t^*BuOH mixture to yield purified peptoid–peptide conjugates as a white powder.

### 3.4. MIC Determination

Compound stocks in DMSO were diluted 50× in cation-adjusted Mueller Hinton broth (CAMHB; 10 mg L^−1^ Mg^2+^, 50 mg L^−1^ Ca^2+^) and serially diluted in polypropylene 96-well plates to reach a volume of 50 µL per well. MRSA USA300 was grown in TSB until the exponential growth phase (OD_600_ = 0.5) before dilution in CAMHB and addition to the wells (50 µL) to reach a final CFU concentration of 5 × 10^5^ mL^−1^. After overnight incubation (35 °C, 250 RPM) the plates were inspected visually for growth. Experiments were carried out in duplicate.

## 4. Conclusions

In conclusion, an efficient and direct methodology that allows the semi-synthesis of novel nisin–peptoid hybrids (**6a** and **6b**) has been developed. The key step in the process involves the use of a Cu(I) catalyzed click reaction. **6a** and **6b** were found to have low micromolar activity against methicillin-resistant *S. aureus* (USA300) with MIC values of 5 µM and 9 µM respectively. This antibacterial activity is similar to full-length nisin (**3**) (MIC 5 µM), and is considerably better than truncated nisin[1,2,3,4,5,6,7,8,9,10,11,12] (**4**) MIC > 100 µM. Given the aforementioned results the primary aim of the work to prepare semi-synthetic nisin analogues with comparable activity to full-length nisin has been achieved. Building on from this proof of concept study we are now expanding the library of nisin–peptoid hybrids, and, looking to investigate in more detail whether this class peptide–peptoid hybrid displays a lipid II targeting mode of action.

## Figures and Tables

**Table 1:** Biological evaluation of peptide–peptoid conjugates and other fragments for comparison against methicillin-resistant *S. aureus* 300. Peptoids and peptide–peptoid hybrids show potent antibacterial action.

	Sequence	MIC (µM)	ED_50_ (μM)	
		*S. aureus* (USA300)	HaCaT	HepG2
**7**	Vancomycin	1		
**3**	Nisin	5		
**4**	Nisin[1,2,3,4,5,6,7,8,9,10,11,12]	>100		
**5**	Nisin[1,2,3,4,5,6,7,8,9,10,11,12]-azide	26		
**1a**	(*N*ae*N*spe*N*spe)_4_	2	26	15
**1b**	[(*N*Lys*N*pfb*N*pfb)(*N*Lys*N*spe*N*spe)]_2_	2–4	53	18
**2a**	*N*prp*N*spe*N*spe(*N*ae*N*spe*N*spe)_4_	4	20	12
**2b**	*N*prp*N*spe*N*spe[(*N*Lys*N*pfb*N*pfb)(*N*Lys*N*spe*N*spe)]_2_	4–7	15	17
**6a**	Nisin[1,2,3,4,5,6,7,8,9,10,11,12]-*N*spe*N*spe(*N*ae*N*spe*N*spe)_4_	5–10	24	15
**6b**	Nisin[1,2,3,4,5,6,7,8,9,10,11,12]-*N*spe*N*spe[(*N*Lys*N*pfb*N*pfb)(*N*Lys*N*spe*N*spe)]_2_	9–18	22	23

## Supplementary Materials

Full materials, synthesis and purification methods, biological protocols, and characterization data for all compounds synthesized are available online.
