# Supplementary material for: Synthesis of Antibacterial Nisin–Peptoid Hybrids Using Click Methodology
Source: Molecules. 2018 Jun 28;23(7):1566. doi: 10.3390/molecules23071566 (PMC6099617; doi:10.3390/molecules23071566)
Supplement: Supplementary file 1 [file molecules-23-01566-s001.pdf]

Supplementary Information for

Synthesis of Antibacterial Nisin-Peptoid Hybrids Using Click Methodology

Hannah L. Bolt,[a,†] Laurens H.J. Kleijn,[b,†] Nathaniel I. Martin[b,*] and Steven L. Cobb[a,*]

[a] Department of Chemistry, Durham University, South Road, Durham, DH1 3LE, UK.

[b] Department of Medicinal Chemistry & Chemical Biology, Utrecht Institute for Pharmaceutical Sciences, Utrecht University, Universiteitsweg 99, 3584 CG Utrecht, The Netherlands.

† These authors contributed equally to this work.

Contents

1 Materials and methods 3
2 Peptoid monomer abbreviations 3
3 Characterisation methods 4
4 Linear peptoid synthesis (1a, 1b, 2a and 2b) 5
5 Digestion of nisin to nisin$^{A/B}$ ring fragment (4) 8
6 Amide-coupled azide-nisin$^{A/B}$ (5) 8
7 Click protocol of alkyne-peptoids with nisin$^{A/B}$-azide (6a and 6b) 9
8 MIC determination 11
9 Toxicity testing 12

1 Materials and methods

All reagents used in this project were purchased from commercial sources and used without further purification unless otherwise specified.m Amines used in submonomer peptoid synthesis were obtained either from Sigma Aldrich (Gillingham, UK) or TCI Europe (Zwijndrecht, Belgium). These chemicals were used without further purification and stored under appropriate conditions, as detailed in the manufacturer's instructions. Bond Elut solid phase extraction cartridges (20 mL, polypropylene with two polypropylene frits) were purchased from Crawford Scientific and used as reaction vessels for solid phase synthesis.

Solvents were removed under reduced pressure using a Büchi Rotavapor R11. A Radleys Discovery Technology shaker was also used to mix solutions where indicated and aqueous solutions were lyophilised using a Christ Alpha 1-2 LD Plus freeze-drier.

Abbreviations for common reagents and protecting groups are as follows: tert-butoxycarbonyl (Boc); trifluoroacetic acid (TFA); triisopropylsilyl (TIPS); *N*,*N*-dimethylformamide (DMF); *N*,*N*-diisopropylcarbodiimide (DIC); dimethylsulphoxide (DMSO); dichloromethane (DCM).

2 Peptoid monomer abbreviations

Monomer	Chemical Structure	Amine Sub-monomer
N**Lys**<br>*N*-(4-aminobutyl) glycine	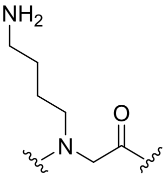	*N*-Boc-1,4-diaminobutane
N**ae**<br>*N*-(2-aminoethyl) glycine	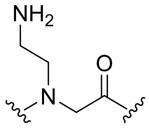	*N*-Boc-1,4-diaminoethane
N**spe**<br>*N*-(*S*)-(1-phenylethyl) glycine	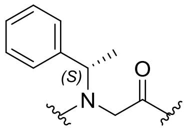	*(S)*-(−)-α-methylbenzylamine
N**pfb**<br>*N*-(4-fluorophenylmethyl) glycine	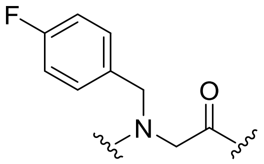	4-fluorobenzylamine
N**prp**<br>*N*-(3-propargyl) glycine	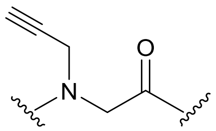	propargylamine

Table S1. Monomer abbreviations, structures and amines used to build the submonomer.

3 Characterisation methods

Quadrupole time of flight accurate mass spectrometry

Accurate mass measurements were performed using a QToF Premier mass spectrometer with an Acquity ultra-performance liquid chromatography system (Waters Ltd, UK). Samples were injected to the Acquity UPLC BEH C18 column (1.7 µm, 2.1 mm x 100 mm) with a flow rate of 0.6 mL min^{-1} and a linear gradient of 0–99 % of solvent B over 6 min (A = 0.1 % formic acid in H_2O, B = 0.1 % formic acid in acetonitrile). The solvent flow from the UPLC was injected into a 0.2 mL/min flow of acetonitrile which was introduced into the electrospray ion source.

Preparative high performance liquid chromatography

Crude peptoids were dissolved into ~1.5 mL (95 % H_2O, 5 % MeCN, 0.1 % TFA) and purified by preparative RP-HPLC using a Perkin Elmer 200 Series LC pump with a Perkin-Elmer 785A UV-vis detector (λ = 250 nm or 220 nm) on a SB Analytical column (ODS-H Optimal), 250 x 10 mm, 5 µm; flow rate = 2 mL min^{-1}; (solvent A = 0.1 % TFA in 95 % H_2O, 5 % MeCN, solvent B = 0.1 % TFA in 5 % H_2O, 95 % MeCN). The gradients chosen were informed by the analytical HPLC retention times of crude products. Relevant fractions were collected, lyophilised and analysed by LC-MS.

Analytical high performance liquid chromatography

Peptoid samples were dissolved in 100 µL acidified water and the purity of products was estimated by an injection of 10 µL to analytical RP-HPLC using a Perkin Elmer 200 Series LC pump with a Perkin-Elmer 785A UV-vis detector on an SB Analytical column (ODS-H Optimal), 4.6 x 100mm , 3.5 µm; flow rate = 1 mL min^{-1}; loop size = 20 µL; λ = 220 nm; gradient: 0–100 % solvent B over 30 min or 60 min (solvent A: 95 % H_2O, 5 % MeCN, 0.05 % TFA; solvent B: 95 % MeCN, 5 % H_2O, 0.03 % TFA).

4 Linear peptoid synthesis (1a, 1b, 2a and 2b)

Modified protocol following the submonomer synthesis of peptoids [H.L. Bolt, S.L. Cobb, *Org. Biomol. Chem.,* **2016**, *14*, 1211]. Fmoc-protected Rink Amide resin (0.1 mmol, loading 0.82 mmol g^{-1}) was swollen in DMF (at least 1 hour, overnight preferred, at room temperature) in a 20 mL polypropylene syringe fitted with two polyethylene frits. The resin was deprotected with piperidine (20 % in DMF *v*/*v*, 2 x 20 min) and washed with DMF (3 x 2 mL). The resin was treated with bromoacetic acid (1 mL, 0.6 M in DMF) and DIC (0.20 mL, 50 % *v*/*v* in DMF) for 20 minutes at room temperature at 400 rpm. The resin was washed with DMF (3 x 2 mL), before the desired amine sub-monomer was added (1 mL, 0.8–2.0 M in DMF) and allowed to react for 60 minutes at room temperature on the shaker. The resin was again washed with DMF (3 x 2 mL) and the bromoacetylation and amine displacement steps were repeated until the final submonomer had been added and the desired peptoid sequence had been obtained.

The resin was shrunk in diethyl ether to remove DMF in preparation for cleavage. Final cleavage from resin was achieved using TFA (95 %), H_2O (2.5 %) and TIPS (2.5 %). For test cleaves approximately 1 mL of the cleavage cocktail was used and for cleavage from 100 mg resin, approximately 4 mL of the cleavage cocktail was added. The resin was then placed on the shaker at 400 rpm for 45 minutes and the resin removed by filtration. The cleavage cocktail was removed *in vacuo*, the crude product precipitated in diethyl ether (45 mL) and the precipitate retrieved by centrifuge for 15 min at 5,000 rpm. The ether phase was decanted, the crude product dissolved in a mixture of acidified H_2O and MeCN and lyophilised. Crude peptoid sequences were purified using RP-HPLC prior to ligation with nisin$^{A/B}$.

	**Sequence**	**Analytical HPLC**		**QToF MS**		**Yield**	
		**RT**	**%**	**Mass calculated**	**Mass observed**	**Mass (mg)**	**Yield (%)**
**1a**	(*N*ae*N*spe*N*spe)$_4$	16.0	>95	$[M+2H]^{2+}$ 853.9847	853.9841	31.9	19
**1b**	[(*N*Lys*N*pfb*N*pfb)(*N*Lys*N*spe *N*spe]$_2$	18.4	>95	$[M+2H]^{2+}$ 917.9971	917.9983	52.0	28
**2a**	*N*prp*N*spe*N*spe(*N*ae*N*spe*N* spe)$_4$	20.0	>99	$[M+2H]^{2+}$ 1062.5873	1062.5857	33.6	32
**2b**	*N*prp*N*spe*N*spe[(*N*Lys*N*pfb *N*pfb)(*N*Lys*N*spe*N*spe)]$_2$	19.0	>99	$[M+2H]^{2+}$ 1126.5997	1126.6017	42.92	38

Table S2. Analytical HPLC and accurate mass spectrometry data for peptoid sequences **1a**, **1b**, **2a** and **2b**.

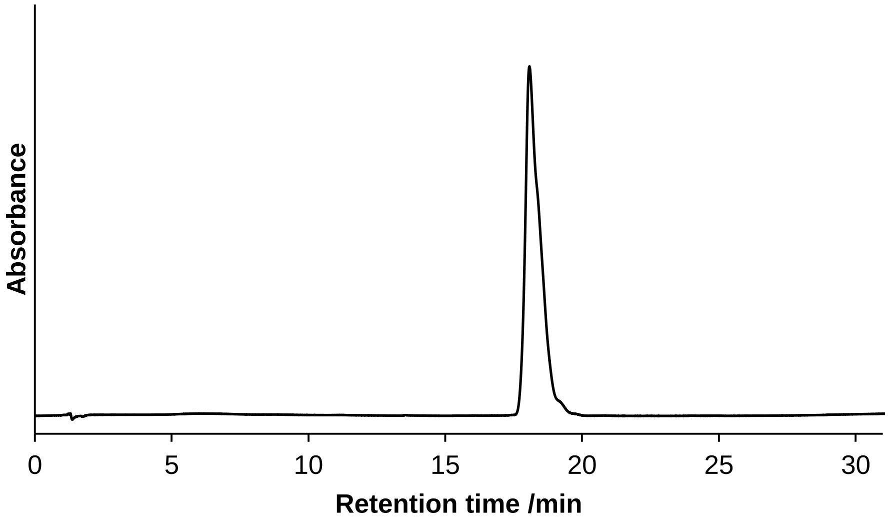

Figure S1. Analytical HPLC trace for peptoid **1a**. λ = 220 nm, flow rate 1 mL min^{-1}, gradient 0–100% B over 30 minutes (where A = 95% H_20, 5% MeCN, 0.1 % TFA; B = 5% H_20, 100% MeCN, 0.1 % TFA).

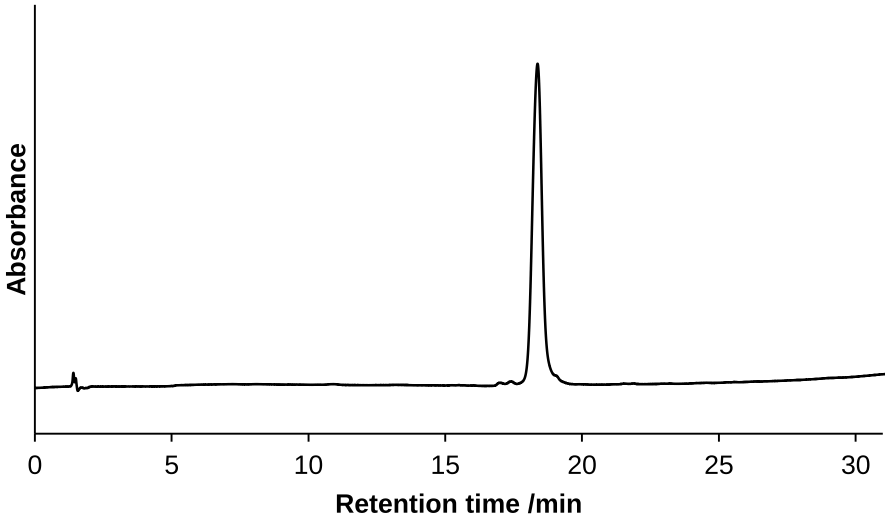

Figure S2. Analytical HPLC trace for peptoid **1b**. λ = 220 nm, flow rate 1 mL min^{-1}, gradient 0–100% B over 30 minutes (where A = 95% H_20, 5% MeCN, 0.1 % TFA; B = 5% H_20, 100% MeCN, 0.1 % TFA).

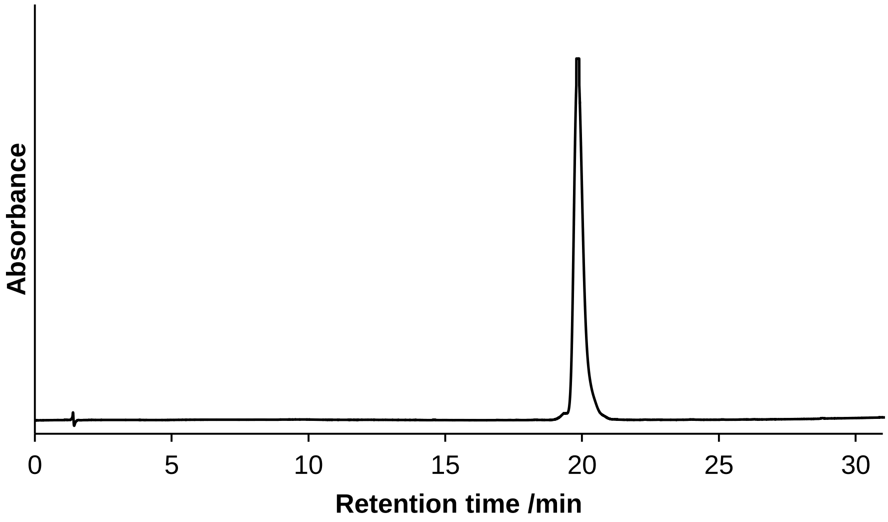

Figure S3. Analytical HPLC trace for peptoid **2a**. λ = 220 nm, flow rate 1 mL min^{-1}, gradient 0–100% B over 30 minutes (where A = 95% H_20, 5% MeCN, 0.1 % TFA; B = 5% H_20, 100% MeCN, 0.1 % TFA).

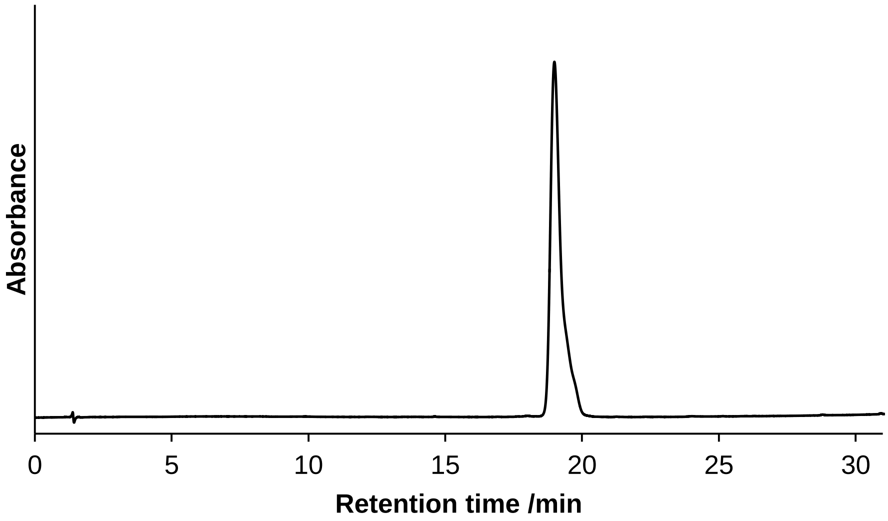

Figure S4. Analytical HPLC trace for peptoid **2b**. λ = 220 nm, flow rate 1 mL min^{-1}, gradient 0–100% B over 30 minutes (where A = 95% H_20, 5% MeCN, 0.1 % TFA; B = 5% H_20, 100% MeCN, 0.1 % TFA).

5 Digestion of nisin to nisin$^{A/B}$ ring fragment (4)

Procedure as in T. Koopmans, T.M. Wood, P. t Hart, L.H.J. Kleijn, A.P.A. Hendrickx, R.J.L. Willems, E. Breukink, N.I. Martin, *J. Am. Chem. Soc.,* **2015**. Nisin (600 mg, 0.18 mmol) was dissolved in 250 mL Tris buffer (25 mmol, NaOAc, 5 mmol Tris acetate, 5 mmol $CaCl_2$, pH 7.0) and the solution cooled on ice for 15 minutes. Trypsin (50 mg) was added and stirred at room temperature for 15 minutes. The mixture was then heated to 30 °C for 16 hours, then another 50 mg of trypsin was added and after an additional 24 hours the reaction was complete by HPLC. The reaction was acidified with HCl (1 M) to pH 4.0 and solvents removed *in vacuo*. The nisin fragment was isolated by preparative HPLC and product fractions lyophilised to obtain a white powder (80 mg, 39 %).

6 Amide-coupled azide-nisin$^{A/B}$ (5)

Procedure as in T. Koopmans, T.M. Wood, P. t Hart, L.H.J. Kleijn, A.P.A. Hendrickx, R.J.L. Willems, E. Breukink, N.I. Martin, *J. Am. Chem. Soc.,* **2015**. Nisin$^{A/B}$ was dissolved in DMF (240 µL). BOP (2 eq.), DIPEA (4 eq.) and azidopropylamine (50 eq.) were added. The reaction was stirred for 20 minutes then quenched in the appropriate buffer (95 % H_2O, 5 % MeCN, 0.1 % TFA, 4 mL). The solution was centrifuged for 5 min at 5,000 rpm to remove any insoluble material and the supernatant was purified by RP-HPLC. Relevant fractions were collected and analysed to yield the pure Nisin$^{A/B}$-azide.

7 Click protocol of alkyne-peptoids with nisin$^{A/B}$-azide (6a and 6b)

10x stock solutions of $CuSO_4$ (16.2 µmol, 2.59 mg in 1 mL H_2O), 10x sodium ascorbate (32.4 µmol, 6.42 mg in 1 mL H_2O) and 10x TBTA (4.1 µmol, 2.18 mg in 1 mL DMF) were freshly prepared. Nisin$^{A/B}$-azide (1 eq., 8.1 µmol, 10 mg) was dissolved in 200 µL DMF and added to the peptoid in the microwave reaction vessel (1 eq., 8.1 µmol). 100 µL of the $CuSO_4$ solution (0.2 eq., 1.62 µmol), 100 µL of sodium ascorbate stock (0.4 eq., 3.24 µmol) and 100 µL of the TBTA solution (0.05 eq., 0.41 µmol) were added. The vessel was sealed and heated under microwave power for 20 minutes at 80 °C. The reaction mixture was diluted in the appropriate buffer (95 % H_2O, 5 % MeCN, 0.1 % TFA, 4mL) and purified by RP-HPLC on a Reprospher 100 C8- or C18- Aqua column (10 µm x 250 x 20 mm) at a flow rate of 6 mL min^{-1}; λ = 214 nm; linear gradient elution 20–80 % solvent B over 120 minutes (where A = 95 % H_2O, 5 % MeCN, 0.1 % TFA; B = 95 % MeCN, 5 % H_2O, 0.1 % TFA). Relevant fractions were combined and lyophilised from 1 : 1 H_2O : ^{t}BuOH mixture to yield purified peptoid-peptide conjugates as a white powder.

Nisin$^{A/B}$-*N*spe*N*spe(*N*ae*N*spe*N*spe)$_4$ (6a)

Nisin$^{A/B}$-azide (8.1 µmol, 10.32 mg in 200 µL DMF) and *N*prp*N*spe*N*spe(*N*ae*N*spe*N*spe)$_4$ (8.1 µmol, 13.89 mg) were conjugated following click protocol above to yield the peptide-peptoid conjugate as a white powder (9.16 mg, 34 %); RP-analytical HPLC RT 33.3 min; approximate purity > 99 %; accurate QToF MS mass calculated $[M+2H]^{2+}$ 1678.4032, mass observed $[M+2H]^{2+}$ 1678.3981.

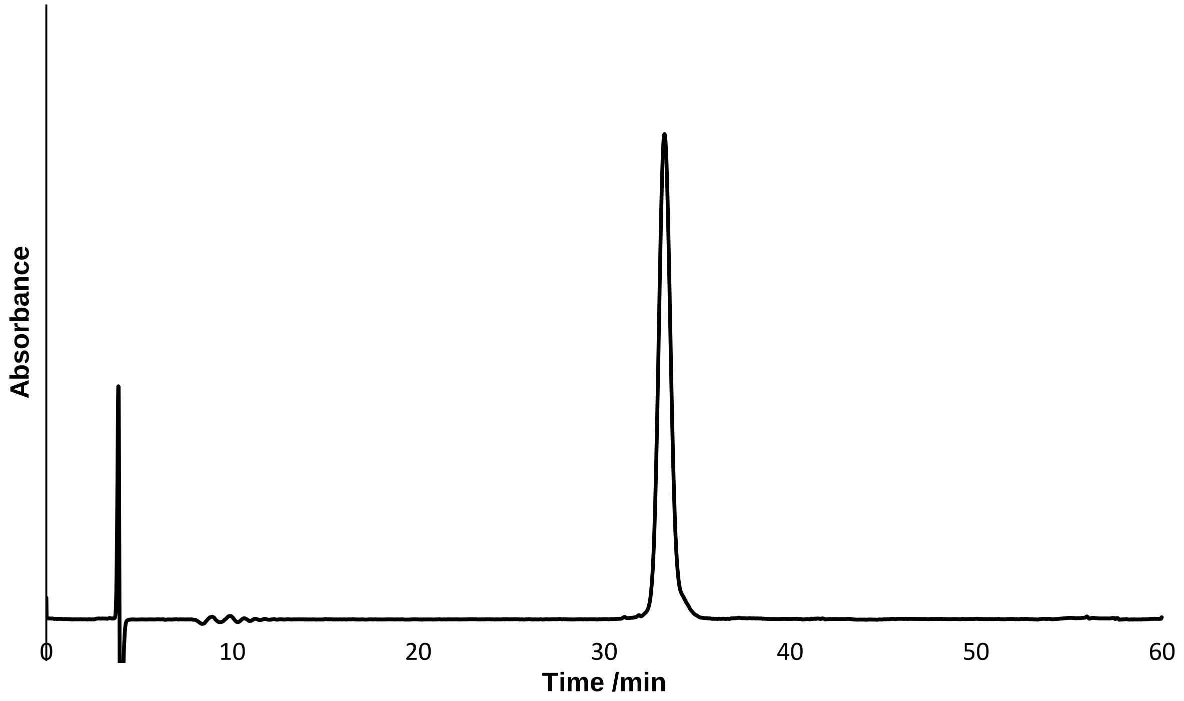

Figure S5. Analytical HPLC trace for peptide-peptoid hybrid **6a**. λ = 220 nm, flow rate 1 mL min^{-1}, gradient 0–100% B over 60 minutes (where A = 95% H_20, 5% MeCN, 0.1 % TFA; B = 5% H_20, 100% MeCN, 0.1 % TFA).

Nisin$^{A/B}$-*N*spe*N*spe[(*N*Lys*N*pfb*N*pfb)(*N*Lys*N*spe*N*spe)]$_2$ (**6b**)

Nisin$^{A/B}$-azide (8.1 µmol, 10.10 mg in 200 µL DMF) and *N*prp*N*spe*N*spe(*N*ae*N*spe*N*spe)$_4$ (8.1 µmol, 18.34 mg) were conjugated following click protocol above to yield the peptide-peptoid conjugate as a white powder (13.86 mg, 49 %); RP-analytical HPLC RT 31.8 min; approximate purity >99%; accurate QToF MS mass calculated $[M+2H]^{2+}$ 1742.4156, mass observed $[M+2H]^{2+}$ 1742.4037.

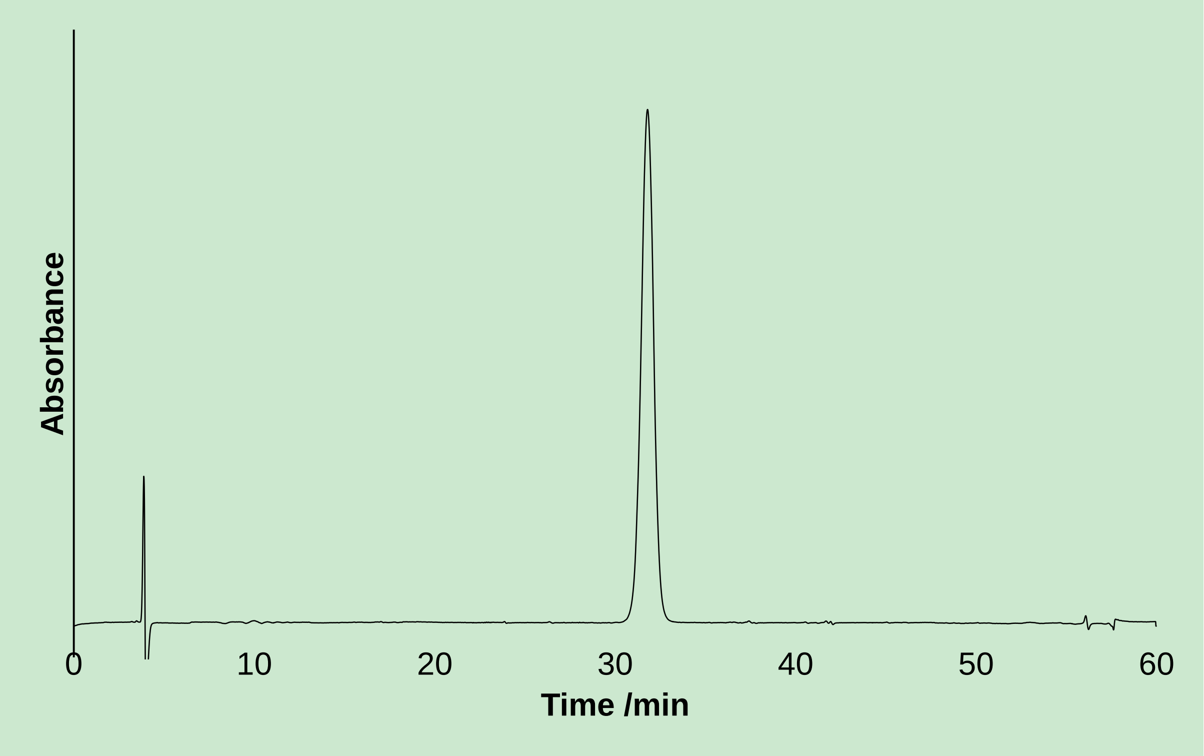

Figure S6. Analytical HPLC trace for peptide-peptoid hybrid **6b**. λ = 220 nm, flow rate 1 mL min^{-1}, gradient 0–100% B over 60 minutes (where A = 95% H_20, 5% MeCN, 0.1 % TFA; B = 5% H_20, 100% MeCN, 0.1 % TFA).

8 MIC determination

Minimum inhibitory concentrations were determined in accordance with CLSI guidelines [CLSI. *Methods for Dilution Antimicrobial Susceptibility Tests for Bacteria That Grow Aerobically; Approved Standard—Ninth Edition.* CLSI document M07-A9. Wayne, PA: Clinical and Laboratory Standards Institute; 2012]. Compound stocks in DMSO were diluted 50x in cation-adjusted Mueller Hinton broth (CAMHB; 10 mg L^{-1} Mg^{2+}, 50 mg L^{-1} Ca^{2+}) and serially diluted in polypropylene 96-well plates to reach a volume of 50 µL per well. MRSA USA300 was grown in TSB until the exponential growth phase (OD_{600} = 0.5) before dilution in CAMHB and addition to the wells (50 µL) to reach a final CFU concentration of $5x10^5$ mL^{-1}. After overnight incubation (35°C, 250 RPM) the plates were inspected visually for growth. Experiments were carried out in duplicate.

9 Toxicity testing

Cytotoxicity analyses were performed in 96-well plates (Costar, Fisher Scientific) using alamarBlue® (Invitrogen) for cell viability detection using a modified protocol as previously described.[9] The HepG2 or HaCaT cells were grown at 37 °C, 5 % CO_2 in $DMEM_{10}$ high glucose supplemented with heat-inactivated foetal bovine sera (FBS, 10 %; Biosera Ltd) and penicillin/streptomycin (P/S, 1 %). Cells were counted using a Neubauer Improved Haemocytometer. Cells were seeded 1 day prior to treatment in 96 well plates at a concentration of 2 x 10^5 cells mL^{-1} in 100 µL of medium (2 x 10^4 cells/well). Empty wells were filled with 100 µL PBS. After 24 hours, cells were incubated with the compounds in a dilution series in triplicate from 2–100 µM (5 mM stock solutions in DMSO; untreated cells with DMSO as a negative control) in 50 µL of the media for 1 hour. Afterwards, 40 µL of medium was removed from each well before the addition of 90 µL of the media, followed by incubation for 24 hours at 37 °C, 5 % CO_2. Then, 10 µL of alamarBlue® (Invitrogen) was added to each well before a 2 hour incubation prior to assessing cell viability using a fluorescent plate reader (Biotek; λ_{ex} 560 nm, λ_{em} 600 nm).

	**Sequence**	**ED_{50} (µM)**		**MIC (µM)**
		**HaCaT**	**HepG2**	***S. aureus* USA300**
**1a**	(*N*ae*N*spe*N*spe)$_4$	26	15	2
**1b**	[(*N*Lys*N*pfb*N*pfb)(*N*Lys*N*pfb *N*pfb)]$_2$	53	18	2–4
**2a**	*N*prp*N*spe*N*spe(*N*ae*N*spe*N* spe)$_4$	20	12	4
**2b**	*N*prp*N*spe*N*spe[(*N*Lys*N*pfb *N*pfb)(*N*Lys*N*pfb*N*pfb)]$_2$	15	17	4–7
**6a**	Nisin[1-12]-*N*spe*N*spe(*N*ae*N*spe*N*spe)$_4$	24	15	5–10
**6b**	Nisin[1-12]-*N*spe*N*spe [(*N*Lys*N*pfb*N*pfb)(*N*Lys*N*spe *N*spe)]$_2$	22	23	9–18

Table S3. Results of MIC determination and toxicity testing against HaCaT keratinocytes and HepG2 epithelial cells.
